# Mapping of Micro-Tom BAC-End Sequences to the Reference Tomato Genome Reveals Possible Genome Rearrangements and Polymorphisms

**DOI:** 10.1155/2012/437026

**Published:** 2012-11-27

**Authors:** Erika Asamizu, Kenta Shirasawa, Hideki Hirakawa, Shusei Sato, Satoshi Tabata, Kentaro Yano, Tohru Ariizumi, Daisuke Shibata, Hiroshi Ezura

**Affiliations:** ^1^Faculty of Life and Environmental Sciences, University of Tsukuba, 1-1-1 Tennodai, Tsukuba 305-8572, Japan; ^2^Kazusa DNA Research Institute, 2-6-7 Kazusa-kamatari, Kisarazu 292-0818, Japan; ^3^School of Agriculture, Meiji University, 1-1-1 Higashi-mita, Tama-ku, Kawasaki 214-8571, Japan

## Abstract

A total of 93,682 BAC-end sequences (BESs) were generated from a dwarf model tomato, cv. Micro-Tom. After removing repetitive sequences, the BESs were similarity searched against the reference tomato genome of a standard cultivar, “Heinz 1706.” By referring to the “Heinz 1706” physical map and by eliminating redundant or nonsignificant hits, 28,804 “unique pair ends” and 8,263 “unique ends” were selected to construct hypothetical BAC contigs. The total physical length of the BAC contigs was 495, 833, 423 bp, covering 65.3% of the entire genome. The average coverage of euchromatin and heterochromatin was 58.9% and 67.3%, respectively. From this analysis, two possible genome rearrangements were identified: one in chromosome 2 (inversion) and the other in chromosome 3 (inversion and translocation). Polymorphisms (SNPs and Indels) between the two cultivars were identified from the BLAST alignments. As a result, 171,792 polymorphisms were mapped on 12 chromosomes. Among these, 30,930 polymorphisms were found in euchromatin (1 per 3,565 bp) and 140,862 were found in heterochromatin (1 per 2,737 bp). The average polymorphism density in the genome was 1 polymorphism per 2,886 bp. To facilitate the use of these data in Micro-Tom research, the BAC contig and polymorphism information are available in the TOMATOMICS database.

## 1. Introduction

Tomato (*Solanum lycopersicum*) is one of the most important vegetable crops cultivated worldwide. Tomato has a diploid (2n = 2x = 24)  and relatively compact genome of approximately 950 Mb [1]. Recently, its genome has been completely sequenced by the international genome sequencing consortium [2].

Genetic linkage maps of tomato have been created by crossing cultivated tomato (*S. lycopersicum*) with several wild relatives, *S. pennellii*, *S. pimpinellifolium*, *S. cheesmaniae*, *S. neorickii*, *S. chmielewskii*, *S. habrochaites*, and *S. peruvianum* [3]. Introgression lines generated from a cross between *S. lycopersicum* and *S. pennellii* have contributed to the isolation of important loci and quantitative trait loci (QTLs) related to fruit size by utilizing DNA markers on the Tomato-EXPEN 2000 genetic map [4–9]. Such interspecies genetic mapping is effective because the divergent genomes provide many polymorphic DNA markers. In contrast, intraspecies mapping is less popular in tomato because of the low genetic diversity within cultivated tomatoes that has resulted from the domestication process and subsequent modern breeding [10]. Recently, we developed SNP, simple sequence repeat (SSR), and intronic polymorphic markers using publicly available EST information and BAC-end sequences (BESs) derived from “Heinz 1706,” a standard line for tomato genomics [11, 12], and applied these markers to create linkage maps between Micro-Tom and either Ailsa Craig, a greenhouse tomato, or M82, a processing tomato, by mapping 1,137 markers [12].

Micro-Tom, a dwarf cultivar, is regarded as a model cultivar for functional genomics of tomato because of several characteristics, including small size (20 cm plant height), short life cycle (3 months), existence of indoor cultivation protocols under normal fluorescent conditions, and high-efficiency transformation methods that have been developed for this line [13–15]. The dwarf phenotype of Micro-Tom is the result of mutations in at least two major recessive loci. *dwarf* (*d*) encodes a cytochrome P450 protein, which functions in the brassinosteroid biosynthesis pathway [16]. Another locus, *miniature* (*mnt*), is suggested to associate with gibberellin (GA) signaling without affecting GA metabolism, but the causal gene has not been identified to date [17]. In Japan, Micro-Tom genomics resources have been extensively accumulated, mainly in the framework of the National BioResource Project (NBRP) (http://tomato.nbrp.jp/indexEn.html). Large-scale ethyl methanesulfonate (EMS) and gamma-ray-mutagenized populations have been created, and visible phenotype data have been accumulated [18–20]. The availability of Micro-Tom genome sequence data will accelerate the mapping of mutant alleles. 

BAC-end sequencing has been performed in the tomato standard line “Heinz 1706” genome project to order BAC clones along the chromosomes [21]. Currently, about 90,000 BESs are available at the Sol Genomics Network (SGN, http://solgenomics.net/). BAC-end sequencing has been conducted for other crop species. In the rice *indica* cultivar “Kasalath,” 78,427 BESs were generated from 47,194 clones and mapped onto the “Nipponbare” reference genome. As a result, 12,170 paired BESs were mapped that covered 80% of the rice genome [22]. Recently, BAC-end sequencing has been performed in crop plants with higher genome complexity. BESs from a commercial sugarcane variety, an interspecific hybrid with complex ploidy, were generated to analyze microsynteny between sugarcane and sorghum [23]. In wheat, which has a complex hexaploid genome, the short arm of chromosome 3A was flow sorted to make a BAC library, and chromosome arm-specific BESs were generated for DNA marker development [24]. In switchgrass, more than 50,000 SSRs were identified from 330,000 BESs, and this enabled detailed analysis on the evolution of this species [25]. A low level of genetic variation has been observed for cultivated peanuts. Polymorphic SSRs were accumulated from the BESs and successfully used in the construction of a genetic map [26]. BAC-end sequencing can be useful as a resource for performing comparative genomic studies through mapping of the sequences to a reference genome and by facilitating the development of polymorphic DNA markers.

In the present study, we generated 93,682 single-pass end sequences from a Micro-Tom BAC library. To compare the structures between the reference tomato “Heinz 1706” genome, mapping of unique ends was performed, and possible genome rearrangements and polymorphisms were identified.

## 2. Materials and Methods

### 2.1. Micro-Tom BAC Library Construction

Micro-Tom (TOMJPF00001) seeds were obtained from the NBRP (MEXT, Japan) and sent to the Clemson University Genomics Institute (CUGI) for BAC library construction. The genomic DNA was partially digested, and fragments were cloned into the *Hin*d III site of pIndigoBAC536. A total of 55,296 clones in *Escherichia coli* DH10B cells were arrayed in 144 384-well plates. 

### 2.2. End Sequencing of Micro-Tom BAC Clones

To analyze BESs, the BAC DNAs were amplified using a TempliPhi large-construction kit (GE Healthcare, UK), and the end sequences were analyzed according to the Sanger method, using a cycle sequencing kit (Big Dye-terminator kit, Applied Biosystems, USA) with a type 3730xl DNA sequencer (Applied Biosystems). The resulting sequence reads were quality checked with PHRED [27, 28], allowing the identification and removal of low-quality (QV < 20) sequences. The 93,682 reads clearing the quality criteria were submitted to DDBJ/GenBank with accession numbers FT227487-FT321168.

### 2.3. Mapping to the Reference Genome and Analyses

BES reads were subjected to similarity search using the BLASTN program [29, 30]. To isolate unique sequences from repetitive ones, 93,682 BESs were searched against the repeat database in ITAG2.3 (http://solgenomics.net/) using a cutoff *E*-value of less than 10^−50^. The remaining sequences were searched against the published version of the “Heinz 1706” genome (SL2.40), which was accessed from the SGN database (http://solgenomics.net/). From all of the BLAST alignments, BESs were extracted according to the following criteria, suggested in a previous report [22]: (1) sequence identity > 90% and alignment coverage > 50%; (2) mapped positions of each pair of ends < 200  kb apart in the same chromosome; (3) direction of each paired end is correct; (4) BLASTN *E* < 10^−100^; (5) a minimum of one hit for one of the paired ends; (6) no redundant chromosomal locations. Sequence polymorphisms (SNPs and Indels) between Micro-Tom and “Heinz 1706” were predicted based on the BLASTN alignment. Since we did not allow a gap exceeding 27 bases, only Indels up to 26 bases in length were counted.

### 2.4. Database and Clone Distribution

Mapped data and SNP/Indel sites were made accessible through the database TOMATOMICS at http://bioinf.mind.meiji.ac.jp/tomatomics/. BAC clones are available upon request from NBRP tomato (http://tomato.nbrp.jp/indexEn.html). 

## 3. Results

### 3.1. General Features of the Generated BESs

**Figure 1:**
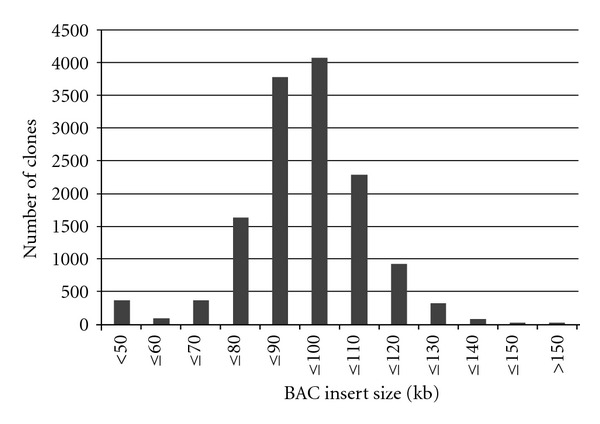
Distribution of BAC clone insert size. The insert size was deduced by mapping BESs onto the reference “Heinz 1706” genome (SL2.40).

The BAC insert size distribution was deduced based on the mapping results. According to these results, 45.4% (6,396 out of 14,101) of the BACs ranged from 100 to 120 kb, with average and median sizes of 101.3 kb and 101.8 kb, respectively (Figure 1). By multiplying by the number of clones (55,296), this BAC library covers 5.9x of the 950 Mb tomato genome.

**Figure 2:**
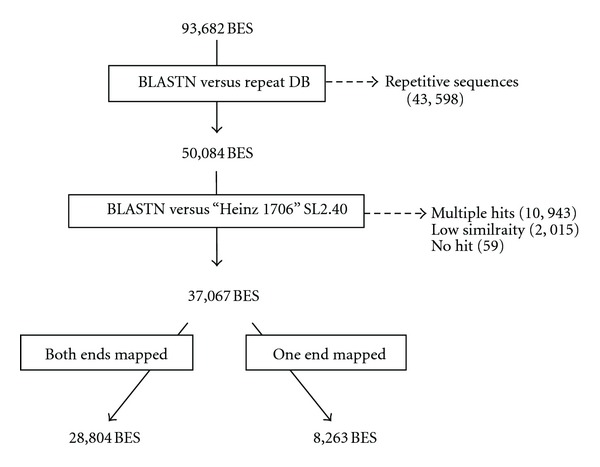
Flow of the BES analysis. To eliminate repetitive sequences, 93,682 BESs were initially searched against the repeat dataset of ITAG 2.3 with a BLASTN cutoff value of *E* <10^−50^. Next, the remaining sequences were mapped onto the “Heinz 1706” pseudomolecule sequences (SL2.40) under the following criteria: identity >90%, coverage >50%; *E* <10^−100^; the inclusion of single hits only. Mapped BESs were classified as either unique pair ends, for which both ends were mapped, or unique ends, for which only one end was mapped.

Micro-Tom BES mapping to the “Heinz 1706” genome was processed as indicated in Figure 2. By eliminating repetitive, redundant, and unmapped sequences, 28,804 “unique pair ends” and 8,263 “unique ends” were selected. Paired-end sequences were mapped onto the reference tomato genome sequence, and 2,248 hypothetical BAC contigs were constructed (see details at TOMATOMICS, http://bioinf.mind.meiji.ac.jp/tomatomics/). The integrity of the hypothetical contigs was confirmed by linking to the DNA markers on two genetic maps, AMF_2_ and MMF_2_ (see Supplementary Table 1 in Supplementary Material available online at doi:10.1155/2012/437026). 

The genome coverage of the hypothetical BAC contigs was assessed by applying euchromatin/heterochromatin boundary information from the genetic map EXPEN2000 [2]. The results indicated that the euchromatin coverage ranged between 45.1% and 71.1% (average, 58.9%) among the different chromosomes, while heterochromatin coverage ranged between 57.4% and 75.3% (average, 67.3%). The total physical length of the BAC contigs was 495,833,423 bp, covering 65.3% of the total chromosomes (Table 1).

### 3.2. Possible Genome Rearrangements

To assess the occurrence of genome rearrangements, Micro-Tom and the reference tomato “Heinz 1706” were compared. Possible inversions, translocations, and insertions were considered. To eliminate an artificial effect (e.g., chimeric BAC clones), only regions covered by more than two BAC clones were selected. After removing regions that had cleared the criteria for extraction (see Section 2) but were either shown to be multicopy by manual evaluation of the BLAST results or displayed similarity to transposable elements, we obtained two cases of a possible rearrangement between Micro-Tom and “Heinz 1706” (Table 2). On chromosome 2, a possible inversion was detected. The size of this inversion could be 20–220 kb depending on which end of the BAC clone is inversed. Translocation and inversion were observed on chromosome 3. For each of two BAC clones (MTBAC041L05 and MTBAC077O14), one of the ends was mapped to 6,601 kb of chromosome 3, while the other end was mapped to 55,665 kb, more than 49 megabases apart. In addition, both ends were mapped on the minus strand.

### 3.3. Polymorphisms between Micro-Tom and the Reference Tomato

SNPs and Indels between Micro-Tom and “Heinz 1706” were identified. Among the SNPs and Indels found, 171,792 were mapped on 12 chromosomes, and 2,635 were mapped on pseudomolecules with no chromosomal information (SL2.40ch00 of the tomato whole-genome shotgun chromosomes) (Table 3 and Supplementary Table 2, see details at TOMATOMICS). According to these results, among the mapped SNPs and Indels, a total of 30,930 polymorphisms were found in the euchromatin (1 out of 3,565 bp), and 140,862 were found in the heterochromatin (1 out of 2,737 bp). The average polymorphism density in the genome was 1 polymorphism per 2,886 bp. Transversion-type SNPs were observed in 83,262 cases, while 60,631 were transition-type SNPs. Among the 30,534 Indels, single-base insertions (on the SL2.40 version of the tomato whole-genome shotgun chromosomes) were observed in 10,740 cases, and single-base deletions were seen in 17,064 cases. The remainder were larger Indels, ranging from 2 to 26 bp (Supplementary Table 2). Classification of polymorphisms regarding genic or intergenic regions is shown in Table 4. 

## 4. Discussion

By selecting unique end sequences from 93,682 reads, 28,804 paired ends (14,402 pairs) and 8,263 unpaired ends were obtained. The majority of the nonselected sequences (43,598) were derived from repetitive regions. For the rest, 10,943 had redundant hits to the “Heinz 1706” genome, possibly including repetitive sequences that were not represented in the repeat database in ITAG2.3 (http://solgenomics.net/), 2,015 showed weak similarity, and 59 showed no similarity (Figure 2). Considering that the genome has been previously estimated to be composed of 25% gene-rich euchromatin [31, 32], BES selection in this study (39.6%, (28,804 + 8,263)/93,682)) could have eliminated repetitive regions to a moderate degree. We identified 59 reads showing no significant similarity to the “Heinz-1706” genome. Micro-Tom was bred by crossing the home-gardening cultivars, Florida Basket and Ohio 4013-3. The pedigree of Ohio 4013-3 suggested that a wild relative species was used in the breeding history [18, 33]. Such introgressed segments may lead to the introduction of genomic regions not harbored by “Heinz 1706.” The Micro-Tom genome is now being sequenced (draft sequence data available at DDBJ with the accession number DRA000311), and mapping of orphan BESs to the *de novo* assembly of Micro-Tom genome data will help to clarify this question. 

The total physical length of Micro-Tom BAC contigs was 495,833,423 bp, which covers approximately 65.3% of the DNA from all 12 chromosomes. In the Kasalath rice BES analysis, chromosomal coverage in relation to the reference Nipponbare pseudomolecule was about 80%, despite the lower number (78,427) of analyzed BESs [22]. Because we used the same criteria for repetitive sequence selection (*E* < 10^−50^), the discrepancy between the two studies might be due to the larger genome size of tomato (950 Mb) compared with rice (430 Mb) [34]. Our Micro-Tom BAC coverage is reasonable, taking into account the scale of the BAC library used.

Micro-Tom has been considered as a model cultivar to promote functional genomics studies of tomato by taking advantage of its characteristics. Currently, many tools and platforms have been developed, and some of these are already available to the research community. The present study characterized the overall polymorphisms found between Micro-Tom BESs and the reference tomato “Heinz 1706” genome. In addition, two possible genome rearrangement events, on chromosome 2 and chromosome 3, were observed (Table 2). In the case of translocation and inversion on chromosome 3, a gene annotated as reverse transcriptase was found in the flanking region (Solyc03g104840.1). We speculate that this region was translocated by the activity of a retrotransposon, as it was in the case of *SUN*. Enhanced expression of *SUN* caused by a gene duplication event mediated by the retrotransposon *Rider* led to an elongated fruit shape [35]. In the future, we plan to sequence the entire BAC and expect that this will help us to characterize these events in more detail. In the case of the other rearrangement possibility, on chromosome 2, we could not find any trace of a retrotransposon. Since these rearrangements took place in euchromatin, which is rich in genes, these regions could represent an interesting target to investigate their possible effects on phenotypic variation between Micro-Tom and the reference tomato.

**Figure 3:**
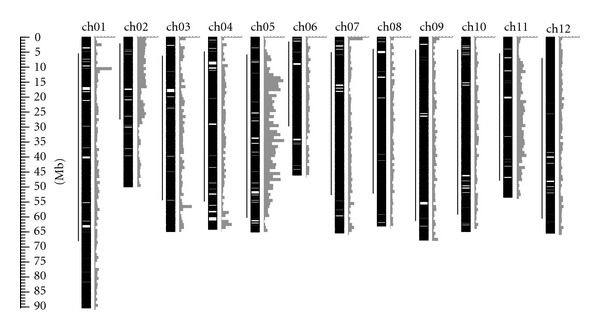
Micro-Tom BAC coverage with respect to the “Heinz 1706” chromosomes and detected polymorphisms. Black boxes indicate covered regions, and white boxes indicate gaps. Bars represent heterochromatic regions. The scale bars for polymorphisms indicate the number of SNPs or Indels per megabase (200 polymorphisms/scale).

We mapped the polymorphisms and depicted them, alongside maps showing covered regions and gaps, in Figure 3. On chromosomes 2, 5, and 11, polymorphisms seemed to be concentrated in the heterochromatic regions; however, this tendency was not clearly observed in the other chromosomes. For the other regions, the polymorphism discovery rate seemed to be somehow correlated with the BAC coverage. Although our analysis indicated little possibility of large-scale genome rearrangement between Micro-Tom and “Heinz 1706” (Table 2), this uneven polymorphism distribution suggests the existence of highly divergent chromosomal regions. The gaps in the hypothetical Micro-Tom BAC contigs could have resulted from low coverage of the BAC library, but the occurrence of chromosomal segments specific to either Micro-Tom or “Heinz 1706” is also possible. The ongoing Micro-Tom genome sequencing and *de novo* assembly of the Micro-Tom genome will clarify the genome structure in detail, enabling a more solid assessment of the differences between Micro-Tom and “Heinz 1706.”

We had previously developed SNP markers among several cultivated tomatoes [12]. By selecting SNPs through *in silico* analysis using public EST information and previously developed SSR markers, 1,137 markers were obtained and successfully mapped on linkage groups between Micro-Tom and either Ailsa Craig or M82. In the present study, we identified 171,792 SNPs and Indels and mapped them on 12 chromosomes. The average density was 1 SNP per 3,565 bp in euchromatin and 1 SNP per 2,886 bp in the genome in general (including both euchromatin and heterochromatin). Previously, large-scale Micro-Tom full-length cDNA analysis and comparison of exon regions with those on the “Heinz 1706” genome revealed a mean sequence mismatch of 0.061% (1/1,640 bp) [36]. One possible explanation for the difference is the quality of the reference “Heinz 1706” genome sequence used in the two studies. We used the published version of the “Heinz 1706” genome sequence, which has higher coverage, giving rise to greater accuracy, although our selection may still contain sequence errors because BESs are single-pass sequences.

The information provided in this study will be useful in the development of DNA markers between Micro-Tom and cultivated tomatoes, which will facilitate a better understanding of the physiological and metabolic differences between them. It would also be useful in the genetic mapping of Micro-Tom mutants through the generation of F_2_ segregating populations.

## Supplementary Material

Supplementary Table 1: The integrity of the hypothetical BAC contigs was confirmed by linking to the DNA markers on two genetic maps, AMF2 and MMF2.
Supplementary Table 2: SNPs and Indels between Micro-Tom and “Heinz 1706” were identified. Among the SNPs and Indels found, 171,792 were mapped on 12 chromosomes, and 2,635 were mapped on pseudomolecules with no chromosomal information (SL2.40ch00 of the tomato whole-genome shotgun chromosomes).

## Figures and Tables

**Table 1:** Coverage of chromosomes by hypothetical Micro-Tom BAC contigs.

		SL2.40ch01	SL2.40ch02	SL2.40ch03	SL2.40ch04	SL2.40ch05	SL2.40ch06	SL2.40ch07	SL2.40ch08	SL2.40ch09	SL2.40ch10	SL2.40ch11	SL2.40ch12	Total (ch01–ch12)
Euchromatin	Chromosome length	27,903,720	24,734,122	16,423,960	13,871,288	10,836,573	17,576,248	17,480,118	15,552,430	10,522,300	9,129,273	11,175,203	12,034,427	**187,239,662**
Euchromatin	no. of Contigs	100	78	45	34	32	52	53	55	37	25	45	44	**600**
Euchromatin	no. of BACs	533	504	401	231	170	339	224	279	176	197	184	317	**3,555**
Euchromatin	Covered bases	17,310,734	14,644,412	11,678,941	6,261,540	5,377,576	10,621,719	8,336,310	9,541,847	6,047,365	5,473,701	6,855,876	8,119,058	**110,269,079**
Euchromatin	Uncovered bases	10,592,986	10,089,710	4,745,019	7,609,748	5,458,997	6,954,529	9,143,808	6,010,583	4,474,935	3,655,572	4,319,327	3,915,369	**76,970,583**
Euchromatin	% Coverage	62.0%	59.2%	71.1%	45.1%	49.6%	60.4%	47.7%	61.4%	57.5%	60.0%	61.3%	67.5%	**58.9%**

Heterochromatin	Chromosome length	62,400,524	25,184,172	48,416,754	50,193,024	54,184,865	28,465,388	47,788,503	47,480,227	57,139,791	55,705,032	42,210,822	53,451,826	**572,620,928**
Heterochromatin	no. of Contigs	175	74	147	131	169	76	135	150	149	159	128	155	**1,648**
Heterochromatin	no. of BACs	1,000	391	903	1,022	752	544	959	856	1,209	1,056	746	992	**10,430**
Heterochromatin	Covered bases	39,941,033	15,405,507	32,458,031	34,993,238	31,099,727	19,672,865	35,988,229	33,980,702	40,376,427	37,964,128	27,534,109	36,150,348	**385,564,344**
Heterochromatin	Uncovered bases	22,459,491	9,778,665	15,958,723	15,199,786	23,085,138	8,792,523	11,800,274	13,499,525	16,763,364	17,740,904	14,676,713	17,301,478	**187,056,584**
Heterochromatin	% Coverage	64.0%	61.2%	67.0%	69.7%	57.4%	69.1%	75.3%	71.6%	70.7%	68.2%	65.2%	67.6%	**67.3%**

Total	Chromosome length	90,304,244	49,918,294	64,840,714	64,064,312	65,021,438	46,041,636	65,268,621	63,032,657	67,662,091	64,834,305	53,386,025	65,486,253	**759,860,590 **
Total	no. of Contigs	275	152	192	165	201	128	188	205	186	184	173	199	**2,248 **
Total	no. of BACs	1,533	895	1,304	1,253	922	883	1,183	1,135	1,385	1,253	930	1,309	**13,985 **
Total	Covered bases	57,251,767	30,049,919	44,136,972	41,254,778	36,477,303	30,294,584	44,324,539	43,522,549	46,423,792	43,437,829	34,389,985	44,269,406	**495,833,423 **
Total	Uncovered bases	33,052,477	19,868,375	20,703,742	22,809,534	28,544,135	15,747,052	20,944,082	19,510,108	21,238,299	21,396,476	18,996,040	21,216,847	**264,027,167 **
Total	% Coverage	63.4%	60.2%	68.1%	64.4%	56.1%	65.8%	67.9%	69.1%	68.6%	67.0%	64.4%	67.6%	**65.3%**

**Table 2:** Possible genome rearrangement events observed in the Micro-Tom and “Heinz 1706” genome.

No.	BAC	End1	Acc	Chr	Direction	From	To	End2	Acc	Chr	Direction	From	To	Possible event
1	MTBAC102D20	T7	FT290741	SL2.40ch02	—	29,374,874	29,375,640	SP6	FT290742	SL2.40ch02	—	29,494,209	29,494,781	*Inversion *
1	MTBAC084K15	T7	FT278701	SL2.40ch02	—	29,375,421	29,376,188	SP6	FT278702	SL2.40ch02	—	29,462,866	29,463,675	

2	MTBAC041L05	T7	FT251747	SL2.40ch03	—	6,601,537	6,602,368	SP6	FT251748	SL2.40ch03	—	55,664,754	55,665,559	*Translocation and Inversion *
2	MTBAC077O14	SP6	FT274148	SL2.40ch03	—	6,602,568	6,603,163	T7	FT274147	SL2.40ch03	—	55,665,296	55,666,020	

**Table 3:** Number of polymorphisms found in each chromosome.

		SL2.40ch01	SL2.40ch02	SL2.40ch03	SL2.40ch04	SL2.40ch05	SL2.40ch06	SL2.40ch07	SL2.40ch08	SL2.40ch09	SL2.40ch10	SL2.40ch11	SL2.40ch12	Total
Euchromatin	no. of polymorphisms	4,152	4,123	3,700	2,863	969	2,417	3,504	2,113	1,932	1,302	1,694	2,161	**30,930**
Euchromatin	Covered bases	17,310,734	14,644,412	11,678,941	6,261,540	5,377,576	10,621,719	8,336,310	9,541,847	6,047,365	5,473,701	6,855,876	8,119,058	**110,269,079**
Euchromatin	kb/polymorphism	4,169	3,552	3,156	2,187	5,550	4,395	2,379	4,516	3,130	4,204	4,047	3,757	**3,565**

Heterochromatin	no. of polymorphisms	12,319	10,694	10,408	9,995	30,951	5,134	8,347	8,562	10,231	9,209	14,937	10,075	**140,862**
Heterochromatin	Covered bases	39,941,033	15,405,507	32,458,031	34,993,238	31,099,727	19,672,865	35,988,229	33,980,702	40,376,427	37,964,128	27,534,109	36,150,348	**385,564,344**
Heterochromatin	kb/polymorphism	3,242	1,441	3,119	3,501	1,005	3,832	4,312	3,969	3,946	4,123	1,843	3,588	**2,737**

Total	no. of polymorphisms	16,471	14,817	14,108	12,858	31,920	7,551	11,851	10,675	12,163	10,511	16,631	12,236	**171,792**
Total	Covered bases	57,251,767	30,049,919	44,136,972	41,254,778	36,477,303	30,294,584	44,324,539	43,522,549	46,423,792	43,437,829	34,389,985	44,269,406	**495,833,423**
Total	kb/polymorphism	3,476	2,028	3,129	3,208	1,143	4,012	3,740	4,077	3,817	4,133	2,068	3,618	**2,886**

**Table 4:** Number of polymorphisms found in genic and intergenic regions in each chromosome.

			SL2.40ch01	SL2.40ch02	SL2.40ch03	SL2.40ch04	SL2.40ch05	SL2.40ch06	SL2.40ch07	SL2.40ch08	SL2.40ch09	SL2.40ch10	SL2.40ch11	SL2.40ch12	Total
Genic	Exon	3′ UTR	115	100	127	108	62	58	59	87	47	15	0	0	**778**
		5′ UTR	157	58	48	23	26	34	28	14	38	2	0	0	**428**
		CDS	1,152	967	1,031	758	820	603	757	605	669	570	662	615	**9,209**
	Intron	Intron	2,035	1,938	2,023	1,971	1,547	1,122	1,778	1,154	1,425	994	1,947	1,172	**19,106**
		Splice junction	19	15	12	13	14	15	13	8	8	7	12	12	**148**

Intergenic			12,993	11,739	10,867	9,985	29,451	5,719	9,216	8,807	9,976	8,923	14,010	10,437	**142,123**

Total			16,471	14,817	14,108	12,858	31,920	7,551	11,851	10,675	12,163	10,511	16,631	12,236	**171,792**

## References

[1] Arumuganathan K, Earle ED (1991). Nuclear DNA content of some important plant species. *Plant Molecular Biology Reporter*.

[2] Tomato Genome Consortium (2012). The tomato genome sequence provides insights into fleshy fruit evolution. *Nature*.

[3] Foolad MR (2007). Genome mapping and molecular breeding of tomato. *International Journal of Plant Genomics*.

[4] Paran I, Goldman I, Tanksley SD, Zamir D (1995). Recombinant inbred lines for genetic mapping in tomato. *Theoretical and Applied Genetics*.

[5] Fulton TM, Nelson JC, Tanksley SD (1997). Introgression and DNA marker analysis of Lycopersicon peruvianum, a wild relative of the cultivated tomato, into *Lycopersicon esculentum*, followed through three successive backcross generations. *Theoretical and Applied Genetics*.

[6] Frary A, Nesbitt TC, Frary A (2000). fw2.2: a quantitative trait locus key to the evolution of tomato fruit size. *Science*.

[7] Liu J, Van Eck J, Cong B, Tanksley SD (2002). A new class of regulatory genes underlying the cause of pear-shaped tomato fruit. *Proceedings of the National Academy of Sciences of the United States of America*.

[8] Cong B, Barrero LS, Tanksley SD (2008). Regulatory change in YABBY-like transcription factor led to evolution of extreme fruit size during tomato domestication. *Nature Genetics*.

[9] Xiao H, Jiang N, Schaffner E, Stockinger EJ, Van Der Knaap E (2008). A retrotransposon-mediated gene duplication underlies morphological variation of tomato fruit. *Science*.

[10] Rick CM, Kesicki E, Fobes JF, Holle M (1976). Genetic and biosystematic studies on two new sibling species of Lycopersicon from interandean Peru. *Theoretical and Applied Genetics*.

[11] Shirasawa K, Asamizu E, Fukuoka H (2010). An interspecific linkage map of SSR and intronic polymorphism markers in tomato. *Theoretical and Applied Genetics*.

[12] Shirasawa K, Isobe S, Hirakawa H (2010). SNP discovery and linkage map construction in cultivated tomato. *DNA Research*.

[13] Meissner R, Jacobson Y, Melamed S (1997). A new model system for tomato genetics. *Plant Journal*.

[14] Emmanuel E, Levy AA (2002). Tomato mutants as tools for functional genomics. *Current Opinion in Plant Biology*.

[15] Sun HJ, Uchii S, Watanabe S, Ezura H (2006). A highly efficient transformation protocol for Micro-Tom, a model cultivar for tomato functional genomics. *Plant and Cell Physiology*.

[16] Bishop GJ, Harrison K, Jones JDG (1996). The tomato Dwarf gene isolated by heterologous transposon tagging encodes the first member of a new cytochrome P450 family. *Plant Cell*.

[17] Martí E, Gisbert C, Bishop GJ, Dixon MS, García-Martínez JL (2006). Genetic and physiological characterization of tomato cv. Micro-Tom. *Journal of Experimental Botany*.

[18] Watanabe S, Mizoguchi T, Aoki K (2007). Ethylmethanesulfonate (EMS) mutagenesis of *Solanum lycopersicum* cv. Micro-Tom for large-scale mutant screens. *Plant Biotechnology*.

[19] Matsukura C, Yamaguchi I, Inamura M (2007). Generation of gamma irradiation-induced mutant lines of the miniature tomato (*Solanum lycopersicum* L.) cultivar ‘Micro-Tom’. *Plant Biotechnology*.

[20] Saito T, Ariizumi T, Okabe Y (2011). TOMATOMA: a novel tomato mutant database distributing micro-tom mutant collections. *Plant and Cell Physiology*.

[21] Mueller LA, Tanskley SD, Giovannoni JJ (2005). The tomato sequencing project, the first cornerstone of the International Solanaceae Project (SOL). *Comparative and Functional Genomics*.

[22] Katagiri S, Wu J, Ito Y (2004). End sequencing and chromosomal in silico mapping of BAC clones derived from an indica rice cultivar, Kasalath. *Breeding Science*.

[23] Silva Figueira TR, Okura V, da Silva FR (2012). A BAC library of the SP80-3280 sugarcane variety (*saccharum* sp.) and its inferred microsynteny with the sorghum genome. *BMC Research Notes*.

[24] Sehgal SK, Li W, Rabinowicz PD (2012). Chromosome arm-specific BAC end sequences permit comparative analysis of homoeologous chromosomes and genomes of polyploid wheat. *BMC Plant Biology*.

[25] Sharma MK, Sharma R, Cao P (2012). A genome-wide survey of switchgrass genome structure and organization. *PLoS One*.

[26] Wang H, Penmetsa RV, Yuan M (2012). Development and characterization of BAC-end sequence derived SSRs, and their incorporation into a new higher density genetic map for cultivated peanut (*Arachis hypogaea* L.). *BMC Plant Biology*.

[27] Ewing B, Hillier L, Wendl MC, Green P (1998). Base-calling of automated sequencer traces using phred. I. Accuracy assessment. *Genome Research*.

[28] Ewing B, Green P (1998). Base-calling of automated sequencer traces using phred. II. Error probabilities. *Genome Research*.

[29] Altschul SF, Gish W, Miller W, Myers EW, Lipman DJ (1990). Basic local alignment search tool. *Journal of Molecular Biology*.

[30] Altschul SF, Madden TL, Schäffer AA (1997). Gapped BLAST and PSI-BLAST: a new generation of protein database search programs. *Nucleic Acids Research*.

[31] Zhong XB, Fransz PF, Eden JWV (1998). FISH studies reveal the molecular and chromosomal organization of individual telomere domains in tomato. *Plant Journal*.

[32] Wang Y, Tang X, Cheng Z, Mueller L, Giovannoni J, Tanksley SD (2006). Euchromatin and pericentromeric heterochromatin: comparative composition in the tomato genome. *Genetics*.

[33] Scott JW, Harbaugh BK (1989). *MICRO-TOM: A Miniature Dwarf Tomato (Circular)*.

[34] Yu J, Hu S, Wang J (2002). A draft sequence of the rice genome (*Oryza sativa* L. ssp. indica). *Science*.

[35] Xiao H, Jiang N, Schaffner E, Stockinger EJ, Van Der Knaap E (2008). A retrotransposon-mediated gene duplication underlies morphological variation of tomato fruit. *Science*.

[36] Aoki K, Yano K, Suzuki A (2010). Large-scale analysis of full-length cDNAs from the tomato (*Solanum lycopersicum*) cultivar Micro-Tom, a reference system for the Solanaceae genomics. *BMC Genomics*.

